# Development of a core outcome set for the evaluation of interventions to enhance trial participation decisions on behalf of adults who lack capacity to consent: a mixed methods study (COnSiDER Study)

**DOI:** 10.1186/s13063-021-05883-5

**Published:** 2021-12-19

**Authors:** V. Shepherd, F. Wood, M. Robling, E. Randell, K. Hood

**Affiliations:** 1 Centre for Trials Research, Cardiff University, Cardiff, UK; 2 Division of Population Medicine, Cardiff University, Cardiff, UK; 3 PRIME Centre Wales, Cardiff, UK

**Keywords:** Informed consent, Proxy, Core outcome set, Consensus methods

## Abstract

**Background:**

Trials involving adults who lack capacity to provide consent rely on proxy or surrogate decision-makers, usually a family member, to make decisions about participation. Interventions to enhance proxy decisions about trial participation are now being developed. However, a lack of standardised outcome measures limits evaluation of these interventions. The aim of this study was to establish an agreed standardised core outcome set (COS) for use when evaluating interventions to improve proxy decisions about trial participation.

**Methods:**

We used established methods to develop the COS including a consensus study with key stakeholder groups comprising those who will use the COS in research (researchers and healthcare professionals) and patients or their representatives. Following a scoping review to identify candidate items, we used a modified two-round Delphi survey to achieve consensus on core outcomes, with equivocal items taken to a consensus meeting for discussion. The COS was finalised following an online consensus meeting in October 2020.

**Results:**

A total of 28 UK stakeholders (5 researchers, 10 trialists, 3 patient/family representatives, 7 recruiters and 3 advisors/approvers) participated in the online Delphi survey to rank candidate items from the scoping review (*n* = 36) and additional items proposed by participants (*n* = 1). Items were broadly grouped into three categories: how family members make decisions, their experiences of making decisions, and the personal aspects that influence the decision. Following the Delphi survey, 27 items were included and ten items exhibited no consensus which required discussion at the consensus meeting. Sixteen participants attended the meeting, including additional patient/family representatives invited to increase representation from this key group (*n* = 2). We reached consensus for the inclusion of 28 outcome items, including one selected at the consensus meeting.

**Conclusions:**

The study identified outcomes that should be measured as a minimum in all evaluations of interventions to enhance proxy decisions about trials. These relate to the process of decision-making, proxies’ experience of decision-making, and factors that influence decision-making such as understanding. Further work with people with impairing conditions and their families is needed to explore their views about the COS and to identify appropriate outcome measures and timing of measurement.

**Trial registration:**

The study is registered on the COMET database (https://www.comet-initiative.org/Studies/Details/1409)

**Supplementary Information:**

The online version contains supplementary material available at 10.1186/s13063-021-05883-5.

## Background

Informed consent is considered a cornerstone of ethically conducted clinical trials. However, some people may have impaired capacity to consent due to acute medical events, or through long-term conditions affecting cognitive function such as dementia, or due to mental illness or learning disabilities, or at the end of life [1–4]. For people who are unable to provide consent, alternative consent processes are required, although these arrangements will differ by jurisdiction [5]. Generally, they provide for an alternative ‘proxy’ or ‘surrogate’ decision-maker to be involved in making a decision about trial participation on the person’s behalf. In England and Wales, research involving adults who lack capacity to consent is governed by the Mental Capacity Act which has provisions for a consultee to provide advice about what, in their opinion, would be the person’s wishes and preferences about participating in the study [6]. The Clinical Trials Regulations, which governs clinical trials of investigational medicinal products including adults lacking capacity, states that a legal representative provides consent that represents the person’s ‘presumed will’ [7]. In the event that no personal consultee or legal representative is available or willing to act as proxy, a professional involved in the care of the person who lacks capacity may be asked to act as nominated consultee or professional legal representative [6, 7]. Proxy decisions about trial participation are complex [8]. As with providing consent for oneself, the consultee or legal representative is usually provided with an information leaflet about the trial and given the opportunity to ask any questions before indicating their agreement by signing a document containing a series of statements [9]. However, unlike conventional consent, consultees and legal representatives are required to represent the person’s wishes and preferences rather than their own. This can be challenging for family members acting as consultee or legal representative, who often experience an emotional and decisional burden as a result [10]. Concerns about the burden of making proxy decisions, together with broader ethical concerns about who should act as proxy and the ethical basis for their decision, contribute to the methodological, structural, and systemic barriers to the inclusion of adults lacking capacity to consent in trials [11].

There has been a growing emphasis on interventions to improve informed consent decisions about whether to participate in a randomised controlled trial (RCT). These interventions primarily focus on the content and process of consent including provision of information and subsequent level of knowledge or understanding of trial information [9]. However, there are also calls for interventions that improve decision-making about participation in clinical trials, beyond solely the ‘informedness’ of potential participants, which enable them to make personally relevant decisions by weighing up what matters most to them within the context of the trial [12]. As a result, there is now a growing interest in interventions to improve the process of decision-making about trial participation, predominantly through the use of decision aids which provide structured guidance on the steps of decision-making to support the decision-making process [13].

### Decision aids for trial participation decisions

Decision aids (DAs) are complex interventions designed to help people make specific, deliberative choices among various options, by providing information about the options and outcomes that are relevant to the decision [14]. There is growing evidence of the effectiveness of DAs in improving both the quality of the decision-making process and decision quality for healthcare decisions [15] and more recently clinical trial participation decisions [13]. Previous studies have highlighted the lack of support for families acting as proxies and have called for interventions to ensure that families have sufficient information and understanding about the role, although the nature of these interventions was not outlined [16].

A novel decision support intervention has now been developed which aims to improve proxy decisions about research on behalf of adults who lack capacity to consent [17]. In order to establish the effectiveness of any such interventions, RCTs or other well-designed studies will be needed [9]. As there are no previous similar interventions, there are no established outcomes or outcome measures for proxy decisions about trial participation. The challenge is to identify what constitutes ‘good’ proxy decisions, in order to effectively measure any improvement in the quality of the decision-making process and the decision itself.

### Core outcome sets for evaluation of DAs for trial participation

When considering outcome selection, researchers must consider an array of information, including how responsive it is to the intervention under investigation, the importance of an outcome to relevant stakeholders, and its acceptability to participants required to complete or measure that outcome [18]. There are well-established methods to guide the development and use of a core outcome set (COS) which is an agreed standardised collection of outcomes which should be measured and reported, as a minimum, in all trials for a specific area [18]. The majority of existing core outcome sets have been developed for the evaluation of interventions in specific clinical conditions, but are now being developed for trial participation decisions [9]. Following the development of the first intervention to enhance proxy decisions about trial participation, this project aimed to utilise similar methods to develop a COS for interventions to support proxy decisions about trial participation, adapted to the context of proxy as opposed to individual decision-making. It built on previous qualitative work to explore proxies’ decision-making processes and decision support needs [10] and was conducted with a range of stakeholders over several phases as outlined below. Details of this study were prospectively registered on the Core Outcome Measures in Effectiveness Trials (COMET) initiative database [19].

## Methods

### Aims and objectives

The aim of this study was to develop a core outcome set for the evaluation of interventions that are intended to improve proxy decisions about trial participation on behalf of adults who lack capacity to consent, using the definition outlined in the Mental Capacity Act 2005 (MCA) [6]. This included all impairing conditions, which may include people living with neurodegenerative conditions such as dementia, resulting from acute medical emergencies or requiring critical care, and people with profound learning disabilities or those requiring palliative care. The term ‘proxy’ was used to refer to those approached to act as a personal consultee [6] or personal legal representative [7]. An outcome domain (or construct) refers to *what* is being measured, and an outcome measure (or instrument) refers to *how* the outcome is being measured [20]. The COS was developed following the approach recommended by the COMET initiative [18] and is reported in accordance with the COS-STAR reporting guidelines [21].

**Fig. 1:**
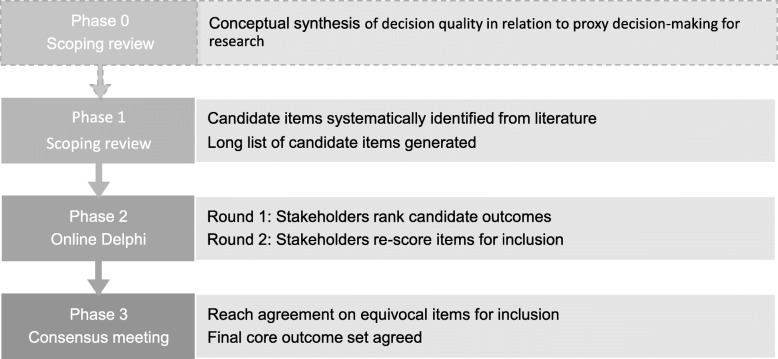
COnSiDER core outcome set development process

For COS development, it is recommended that potentially relevant outcomes are identified from existing work to inform the consensus process, including from systematic reviews of published studies [18]. As this is a novel area of research, a concept synthesis [manuscript currently under review] was first conducted to explore the conceptual aspects of decision quality relating to proxy decision-making for research and to identify candidate outcome domains using an approach proposed by Walker and Avant [22]. In this phase (Phase 0), literature was reviewed from the areas of decision science and decision support, informed consent, and proxy decision-making to explore what are considered to be the constituents of proxy decision-making (i.e. the process) and decision quality (i.e. the decision itself) and identify domains which captured these aspects. The concepts informed the search strategy for a scoping review and provided context within which the outcomes could then be identified and considered for inclusion in the COS. An overview of the COnSiDER COS development process is shown in Fig. 1.

### Phase 1: Scoping review

The scoping review [23] was conducted to identify candidate outcomes used in trials of relevant decision support interventions. This scoping review broadly followed the methodological framework outlined by Arksey and O’Malley using a number of stages: identifying the research question, identifying relevant studies, study selection, charting the data, and collating, summarising, and reporting the results [24] and informed by more recent enhancements to the framework and recommendations for its use proposed by Levac et al. [25]. It is reported in accordance with the PRISMA guidance [26].

#### Search strategy

In scoping reviews, study selection is considered an iterative process involving searching the literature, refining the search strategy, and reviewing articles for study inclusion [25]. The search strategy was iteratively developed. As this is a novel area of research, the search terms were adapted from that used in a recent Cochrane review of DAs for trial participation decisions [13]. This included conducting an update of the Cochrane review search (from 2015 to current) and expanded to include terms relating to ‘proxy’ or ‘surrogate’ decisions (see Additional file 1: Appendix 1. Search strategy for DAs for proxy decisions). The searches were conducted in August 2019. Initial terms used the key elements of proxy or surrogate (population), decision-making, decision aids, or decision support (concepts), and informed consent, research, or clinical trials (context). Bibliographic databases were searched, including MEDLINE, EMBASE, and PsycINFO. The search was not limited by date but was limited by including only English language papers and published studies that had full text available (e.g. ‘grey’ literature and conference abstracts were excluded). Supplementary searches were conducted, including citation tracking, reference lists of included papers, and electronic table of contents of key journals (e.g. BMC Medical Informatics and Decision Making) for the last 2 years.

#### Inclusion and exclusion criteria

Included studies were evaluations of decision support interventions to improve consent in trials or proxy decision-making for care/medical treatment. These included randomised controlled trials, mixed-methods, qualitative studies, questionnaires, surveys, focus groups, and other methods. In addition to publications reporting completed studies, protocols for prospective evaluations of decision aids were also included. Decision aids were those meeting the criteria of a decision aid as determined by the International Patient Decision Aid Standards (IPDAS) Collaboration [27]. Studies that explored decision support interventions for general decision-making (i.e. not using a proxy) for healthcare, treatment, or health screening decisions were excluded. Discussion papers, conference abstracts, and systematic reviews were also excluded. Citations identified through the search were screened and assessed for inclusion by one researcher (VS) and independently assessed by a second reviewer (ER) to ensure validity of the application of the eligibility criteria. A quality assessment of the methodological limitations or risk of bias of the evidence was not conducted as it is generally not performed in a scoping review [28].

#### Data extraction

Data were extracted independently by two researchers (VS and ER) using a data extraction form designed for the review and piloted prior to use. Extracted data included details of the author, year and journal of publication, study type, study aim, host study context (for example, condition, trial design and intervention(s)) where relevant, outcome domains assessed, and any outcome measurement instruments used. The data on outcomes measures was not included in Phase 2 but will inform future work to develop a core measurement set.

#### Data analysis

Following data extraction, data were summarised and presented in tabular form. Details of all outcome domains and measures, and the relevant studies they were identified through, were recorded. Outcome measures were mapped against the outcome domains identified, and any areas of absence (i.e. where there were no outcome measures found for domains identified as relevant in the scoping review or literature review) highlighted. Where outcomes were considered to be duplicates, they were combined, and any included outcomes considered not to be relevant during the data analysis stage were removed (with reasons provided). The findings were analysed in relation to the research questions and overall study purpose. The list of candidate domains was then taken forward to the next phase of the study for consultation with stakeholders and the outcome measures were held separately for future work to establish which measures captured the outcome domains to be established during this project.

### Phase 2: Consultation with stakeholders

A consultation exercise was conducted to identify an agreed set of outcomes (COS) to represent the minimum that should be measured and reported in trials of interventions to improve proxy decisions about research on behalf of adults who lack capacity to consent. In COS development, diverse stakeholder involvement in reaching a consensus is seen as one of the essential collaborative components [18]. For this project, stakeholders were invited to participate in an online Delphi survey followed by a final consensus meeting.

#### Participant identification and recruitment

A broad range of stakeholder groups were invited to participate in the consultation exercise, including:

• People with experience of living with a condition that may impair decisional capacity

• Family members of people with impairing conditions (including those who have had experience of acting as a proxy)

• Researchers who design and conduct trials with adults who lack capacity

• Research nurses involved in recruiting adults who lack capacity

• Researchers engaged in communication and decision-making research

• Ethicists and methodologists with an interest in trials and/or informed consent.

Participants were recruited through existing research networks, trials methodology networks, and using social media (Twitter).

The Delphi method (including eDelphi which uses online communication) is a well-recognised approach for gaining consensus among a group of experts and is commonly used in COS development [18]. There is currently no standard method for determining sample size calculations for Delphi studies. However, there is emerging evidence in the literature that expert panels of around 20 can provide stable results [29]. As proxy decision-making for research is a relatively under-researched area, the number of stakeholders engaged and informed about the topic was expected to be lower than other condition-specific core outcome set development groups, or those to develop a core outcome set for the evaluation of interventions intended to improve informed consent for randomised controlled trials [9].

#### Ethical considerations

Ethical approval was obtained from Cardiff University School of Medicine Research Ethics Committee prior to commencing the online survey. Participants were asked to tick a box at the start of the survey (both Round 1 and Round 2) to confirm that they consented to participate.

#### Data collection

Data collection was via a web-based system designed to facilitate COS development (DelphiManager, supplied and managed by COMET [30]). Participants who agreed to participate registered their details online and provided demographic data including specifying which stakeholder group they consider themselves to be associated with. Once registered, they were allocated a unique participant ID.

Each outcome domain was represented by a Delphi survey item. The long list of 36 candidate items identified during the scoping review were broadly categorised into three areas: how family members make decisions (e.g. deliberation processes), their experiences of making decisions (e.g. feeling satisfied), and the personal aspects that influence the decision (e.g. being informed). These are listed in Additional file 2: Appendix 2.

##### Round 1

In Round 1, participants were provided with a list of items for scoring grouped into the three categories, alongside a definition of each of the items to aid participant understanding. The respondents were asked to consider the following:

‘Think about the process of making a decision on behalf of an adult who lacks capacity to consent to take part in a clinical trial. How important do you think each item listed below would be in judging how well that decision-making process had been conducted?’

In line with a previous Delphi survey for developing a core outcome set for interventions to improve decisions about participating in clinical trials [9], participants were asked to score each of the listed items using the Grading of Recommendations, Assessment, Development and Evaluations (GRADE) scale of 1 to 9 [31]. The scale was annotated to illustrate that a score of 1 to 3 is interpreted as having ‘limited importance’, 4 to 6 as ‘important but not critical’, and 7 to 9 as ‘critical’ [31]. Participants were instructed to rate all items on their own merit even if they appeared similar. An explanatory ‘help’ text could be accessed for each item which included a further description of the item. A free text box was provided for participants to suggest any additional outcomes. Any new additional outcomes listed by participants were reviewed to ensure they were distinct from those listed. All items (including any new outcomes identified in Round 1) were retained and carried forward to Round 2.

##### Round 2

In the second round, participants were presented with the list of items and definitions, alongside the distribution of scores for each item. Participants were then asked to rescore all items and consider whether they should be included in a core outcome set, with a text box provided for an explanatory comment.

#### Data analysis

Descriptive statistics were used to summarise the number of participants who scored each item and the distribution of scores, alongside the number of respondents who scored the items across both rounds. For each item, the proportion of respondents scoring 1-3, 4-6 and 7-9 on the scale was calculated. In line with previous Delphi surveys [9, 18], each item was classified as follows: ‘consensus in’ (that is, consensus that the outcome domain should be included in a core set), ‘consensus out’ (that is, consensus that the outcome domain should not be included in a core set), or ‘no consensus’ (that is, items that were equivocal and required further clarification), according to the classifications in Table 1.

**Table 1:** Definition of consensus

Consensus classification	Description	Definition
*Consensus in*	Consensus that outcome should be included in the core outcome set	≥ 70% scoring 7 to 9 AND < 15% scoring 1 to 3
*Consensus out*	Consensus that outcome should not be included in the core outcome set	≥ 70% scoring 1 to 3 AND < 15% scoring 7 to 9
*No consensus*	Uncertainty about importance of outcome	Anything else and no new compelling reasons in the comment boxes regarding why

### Phase 3: Consensus meeting

The final phase of the stakeholder consultation was a meeting with participants from the Delphi survey to reach consensus on the items to be included in the core outcome set. Public and patient involvement is a key element in COS development. As members of the public (including patients and families) were under-represented in the Delphi group, additional public contributors were invited from a lay advisory group who support a wider programme of research exploring the challenges of impaired capacity and consent in trials. The main aim of the consensus meeting was to determine consensus (in or out) for those items that were equivocal and did not reach consensus following the online Delphi survey. Prior to the meeting, a briefing summary was provided to participants which included the items that had reached consensus and those for discussion at the meeting. The results from the Delphi survey were presented during the meeting. Items for which there was disagreement in Round 2 were the focus of the discussions aimed at agreement on the final list of outcomes. Due to coronavirus restrictions in place at the time, the consensus meeting was held remotely using a video conferencing platform (Zoom) and on-screen polling. Following discussion of each item, participants were asked to vote to either include or exclude the item.

## Results

### Phase 1: Scoping review

**Fig. 2:**
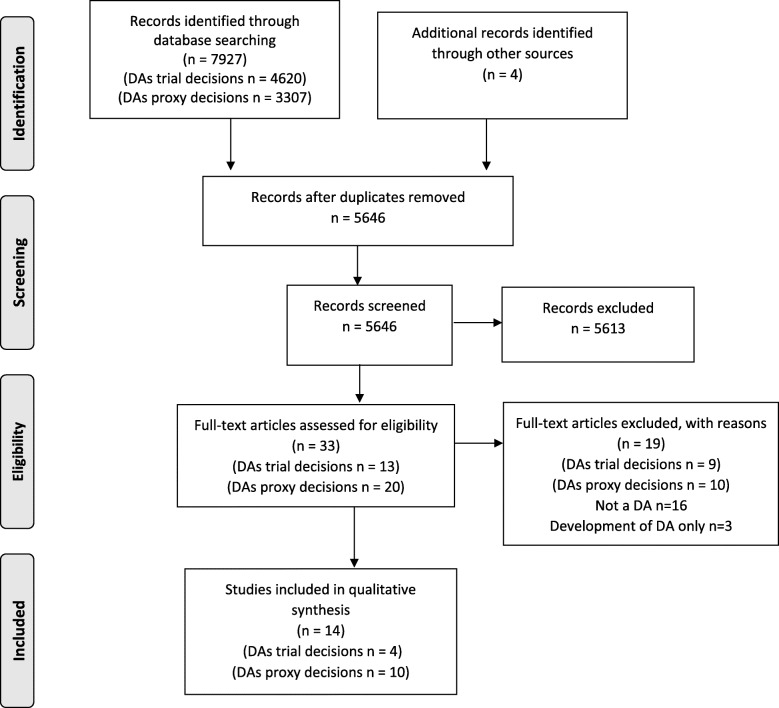
PRISMA diagram of studies included in the scoping review

The Preferred Reporting Items for Systematic Reviews and Meta-Analyses (PRISMA) diagram (Fig. 2) illustrates the flow of studies throughout the review. The searches identified 7927 papers which were screened with 33 assessed for inclusion. Of those, 19 were excluded following full text review with 14 meeting the criteria for inclusion. Of the studies that were included, the majority reported DAs for other proxy decisions (*n* = 10) [32–41] with the remainder reporting DAs for trial participation decisions (*n* = 4) [42–45] from the Cochrane review [13] and updated search. The characteristics of the included studies are listed in Table 2.

**Table 2:** Characteristics of studies included in the scoping review

Lead author name	Publication date	Setting	Type of decision	Self or proxy decision	Outcome domains
Juraskova et al. [42]	2015	Oncology Australia and New Zealand	Participation in breast cancer trial	Self	Anxiety/depression; attitudes towards participating; decisional conflict; involvement preferences; actual (objective) understanding; perceived (subjective) understanding
Politi et al. [43]	2016	Oncology USA	Participation in cancer trial (multiple cancers and trials)	Self	Clarity of opinion about participating; decision readiness; decisional conflict; intent to participate; knowledge; self-efficacy
Sundaresan et al. [44]	2017	Oncology Australia and New Zealand	Participation in prostate cancer trial	Self	Anxiety/depression; decisional regret; decisional conflict; knowledge
Robertson et al. [45]	2019	Oncology Australia	Participation in acute lymphoblastic leukaemia trial (children and young people)	Self	Acceptability of DA; decisional conflict; emotional safety; feasibility; involvement in decision-making; knowledge
Cox et al. [32]	2012	Intensive care USA	Prolonged mechanical ventilation provision in critical illness	Proxy	Acceptability of DA; conflict with physicians; decisional conflict; feasibility; physician-surrogate discordance; quality of communication; trust in physician; comprehension of relevant information
Einterz et al. [33]	2014	Nursing homes USA	Treatment decisions for person with advanced dementia	Proxy	Clinician–surrogate concordance; involvement in decision-making; knowledge; quality of communication; satisfaction with care
Hanson et al. [34]	2011	Nursing homes USA	Feeding options in advanced dementia	Proxy	Clinician–surrogate concordance; decisional regret; frequency of communication with health care providers; involvement in decision-making; knowledge
Snyder et al. [35]	2013	Nursing homes USA	Feeding options in advanced dementia	Proxy	Decisional conflict; knowledge
White et al. [39]	2012	Intensive care USA	Decisions about treatment options in critical illness	Proxy	Acceptability of DA; decisional confidence; feasibility; perceived effectiveness of DA; quality of communication; self-efficacy
Cox et al. [40]	2019	Intensive care USA	Decision about prolonged mechanical ventilation provision in critical illness	Proxy	Anxiety/depression; clinician–surrogate concordance; decisional conflict; perception of care centeredness; quality of communication; comprehension of relevant information
Hanson et al. [41]	2017	Nursing homes USA	Treatment decisions for person with advanced dementia	Proxy	Advance Care Planning problem score; satisfaction with decision; decisional conflict; involvement in decision-making; quality of communication; satisfaction with care
Lord et al. [36]	2017	Memory clinics UK	Dementia family carers deciding about place of care	Proxy	Acceptability of DA; anxiety/depression; decisional conflict
Malloy-Weir and Kirk [37]	2017	Nursing homes Canada	Initiation of antipsychotic medications for person with dementia	Proxy	Satisfaction with decision; decisional conflict; knowledge
Mitchell et al. [38]	2001	Acute care Canada	Placement of a percutaneous endoscopic gastrostomy tube for older adult > 65 with cognitive impairment	Proxy	Acceptability of DA; decisional conflict; knowledge

The median number of outcomes included in the studies was 5 (range 2–8). As the populations, settings, and aims of the decision aids (DAs) were diverse, the outcomes used in included studies were similarly varied. Some outcomes related specifically to the intervention rather than decision-making, e.g. acceptability and feasibility of the DA, Advance Care Planning score. However, almost all studies included outcomes relating to participant knowledge and understanding (*n* = 9). Decisional conflict was commonly used as an outcome (*n* = 11), with few using decision regret (*n* = 2) or satisfaction with the decision (*n* = 2). Some studies included measures of decision-maker involvement (or involvement preferences) (*n* = 5) and a similar number measuring quality of communication (*n* = 5) although these were all in the studies exploring proxy decision-making.

The outcomes were reviewed in relation to the research questions and the concept synthesis (manuscript in preparation) of the relevant literature and empirical studies. This enabled the outcomes to be expanded and refined to encompass both decision-making about trial participation (rather than clinical decisions) and proxy decisions (in contrast with participation for oneself). Outcomes relating to the specific intervention rather than decision-making were removed. This resulted in a ‘long list’ of 36 candidate items which formed the basis for the stakeholder consensus consultation (Additional file 2: Appendix 2).

### Phase 2: Consultation with stakeholders

The online Delphi was conducted over two rounds between 12 March and 29 July 2020. A total of 28 participants from across the UK completed the online survey. Participant characteristics are presented in Table 3 and reported for each round. They included family members of people with impairing conditions such as dementia, researchers in a range of relevant areas, trial designers and clinicians leading trials in areas such as emergency care, trial authorisers or advisors (e.g. members of public and patient involvement (PPI) groups and members of Research Ethics Committees), and trial recruiters (e.g. research nurses). In Round 1, two participants provided baseline data only and one did not provide scores for all candidate items. Of the 22 who participated in Round 2, three did not provide scores for all candidate items.

**Table 3:** Participant data

		Participants recruited (***n*** = 28)	Completed Round 1 (***n*** = 26)^**a**^	Completed Round 2 (***n*** = 22)^**b**^
**Age**				
	18–24	0	0	0
	25–34	6	5	5
	35–49	14	13	10
	50–65	3	3	3
	65+	5	5	4
**Gender**				
	Male	10	9	8
	Female	18	17	14
	Other (please specify)	0	0	0
**Country**				
	England	20	19	15
	Northern Ireland	1	1	1
	Scotland	1	1	1
	Wales	6	5	5
**Principal stakeholder group**				
	A family member/friend of someone affected by impaired capacity	3	3	3
	A researcher interested in aspects of trial design and conduct (e.g. ethicist, sociologist)	5	5	4
	A trial authoriser or advisor (e.g. research ethics committee member, PPI partner)	3	3	3
	A trial designer (e.g. trialist, methodologist, lead clinician)	10	9	7
	A trial recruiter (e.g. research nurse)	7	6	5

^a^Participants with missing data *n* = 1

^b^Participants with missing data *n* = 3

During Round 1, six additional outcomes were proposed by three participants (see Additional file 2: Appendix 2). These were reviewed by members of the project team (VS, FW, MR, KH) who determined that only one item was both relevant to the aim of the COS and distinct from those already listed. In Round 2 participants re-scored the list, including the one additional item (see Additional file 3: Appendix 3). There were 67 changes to scores by 16 participants, with 47 scores increased and 20 that were revised downwards. Reasons given for the changes were that participants felt they had over scored the item in the previous round or now felt it was less important; had misread the questions; were influenced by the percentages answered by other participants in the previous round; or had reflected further or had additional experience since completing the previous round. Following Round 2, 27 items reached consensus for inclusion and there was no consensus reached for 10 items which were taken forward to the consensus meeting for discussion.

Of the stakeholders who participated in the online survey, 20 registered to take part in the online consensus meeting held via Zoom in October 2020. However, as this coincided with a ‘second wave’ of COVID-19 some participants who provide clinical services were unable to attend on the day. Sixteen participants attended the consensus meeting, including representation from England and Wales and across the five stakeholder groups, also including additional patient/family representatives who were invited to increase representation from this key group (*n* = 2). Each of the 10 items was presented and discussed by the group in turn, followed by on-screen polling. Participants were asked to vote on whether the item should be included or not in the final COS. A revised threshold for consensus was used in this final meeting to that used in the online rounds, with 70% of those voting needing to agree to its inclusion.

The polling scores and consensus status of items that were considered at the consensus meeting are listed in a supplementary file (Additional file 4: Appendix 4). The results of the polls demonstrated the range of views held by the meeting participants regarding some items. Other items were closely matched in terms of the number of participants who voted for their inclusion or exclusion (items no. 6 and 9), a small number received almost unanimous votes for exclusion (items no. 3 and 10), others achieved a clear majority for inclusion but did not reach the pre-defined threshold. Of the 10 items needing consensus and discussed at the final meeting, only one (item no.2) was included. The 28 items reaching consensus for inclusion in the final COS following the Delphi survey and consensus meeting are shown in Table 4 with additional explanatory terms in italics where appropriate. The use of the term ‘person’ refers to the person lacking capacity to consent that the proxy is making a decision on behalf of.

**Table 4:** Items included in the COnSiDER core outcome set

**How the proxy makes the decision**	
Whether the proxy decision-maker:	
Makes a decision that fits with the person’s own values, wishes, and preferences *(values congruence)*	
Is able to determine the person’s own values, wishes and preferences about the choices	
Is clear about which risks/side-effects of the study would matter most to the person	
Is clear about which benefits from the study would matter most to the person	
Is clear about which benefits or risks/side-effects from the study would be more important to the person *(balancing benefits and risks)*	
**Experiences of decision-making in this context**	
Whether the proxy decision-maker:	
Feels it was the right decision	
Feels satisfied with the decision	
Feels that they had enough time to make a decision	
Feels that the decision process was good (regardless of the outcome)	
Is comfortable with the decision	
Feels that the decision was a wise one	
Feels that they have enough support from others to make a decision	
**Personal characteristics that influence the decision**	
Whether the proxy decision-maker:	
Recognises that a decision needs to be made *(choice awareness)*	
Has been informed about the purpose of the study, procedures, risks and benefits	
Has been informed about their role in making the decision	
Understands that the person’s own values, wishes, and preferences affect the decision	
Understands the information needed to make the decision *(objective understanding)*	
Feels that they understand the information well enough to make the decision *(subjective understanding)*	
Recognises they do not have enough information about the views of the person to represent their views	
Feels confident in their knowledge to make a decision	
Feels confident to make a decision	
Feels able and has the opportunity to ask questions	
Feels able to express their opinion about each choice	
Feels as involved in the decision as they want to be	
Feels prepared to make the decision	
Is ready to make a decision	
Feels that they can delay their decision if they feel that they need more time	

## Discussion

A set of outcomes for the evaluation of interventions to improve proxy decisions about trial participation on behalf of adults who lack capacity to consent has been established using recognised COS development methods. As a minimum, this set of outcomes should be measured and reported in all trials evaluating such an intervention [18]. The aim of developing a COS at an early stage in this novel methodological area is to improve the design and conduct of future studies, and subsequently improve the interpretation and comparison between studies by minimising heterogeneity and reducing the potential risk of outcome selection and reporting bias. The findings will be submitted to the database of the Core Outcome Measures in Effectiveness Trials initiative (COMET )[30]. Whilst there is an expectation that all the core outcomes will be collected and reported, the outcomes in a particular trial may not be restricted to only those in the COS. As a novel and developing methodological area, researchers may continue to explore other outcomes as greater knowledge and understanding about this topic is gained.

The complex concepts involved in proxy decision-making for research were highlighted by participant comments from the Delphi survey and discussions at the consensus meeting. A range of views were expressed during the meeting about the importance of each item, which echoed the lack of consensus reached during the online survey rounds. Discussions centred around the complexity of decision-making and proxy consent decisions made on behalf of others, and the overlapping relationships between many of the outcomes (e.g. the role of regret about decision-making vs regret about the decision made vs role regret). As reflected in the literature, it is unclear how much these should be considered features of decision-making rather than problematic areas that should be targeted for reduction [46]. There were also particular discussions around differentiating the *process* of decision-making from the *outcomes* of decision-making and how these are experienced in practice. Following on from determining the outcomes to be included in the COS, discussions in the consensus meeting moved onto further questions about how (and particularly when) the outcomes should be measured.

### Methodological considerations and strengths and limitations

As a novel area for research, the complex concepts around enhancing decision-making about research on behalf of someone else have had limited exploration both theoretically and empirically. This can present challenges when involving a range of stakeholders to achieve consensus around the concepts involved. Several participants commented during the online Delphi rounds that it was a challenging exercise. They also commented that participating had encouraged them to think about research involving adults lacking capacity and the whole framework of mental capacity and decision-making in far greater depth than previously. However, it may have been difficult for participants to differentiate between matters of process in decision-making and outcomes of decision-making, and there may have been perceived overlap between the candidate items. For example, three of the items discussed at the consensus meeting concerned the concept of regret. None of these reached the threshold for inclusion, although ‘Has feelings of regret about the decision’ (item no. 7) came close, despite decisional regret being a common outcome in DAs for other types of decisions [13]. This exclusion may be due to the widely recognised complexity around the multiplicity of regret types (process, option, and outcome regret) [46] or that participants considered regret to be a less important outcome in the context of proxy decisions about participation than those selected for inclusion.

What was considered to be expected or ‘normal’ in this decision-making context was the subject of much debate, and the importance of timing in regard to when outcomes should or could be measured. Unlike the well-trodden discussions and widely understood concepts that are the focus of condition-specific core outcome sets, this COS has simultaneously explored what a quality proxy decision looks like in practice, and how it should be measured. This lack of a well-established understanding of the phenomenon, and associated uncertainty, may have been reflected in the low numbers of candidate items that were scored as ‘not important’ by participants. The high threshold for ‘consensus out’ meant that many items did not reach this and has been identified as an issue in other COS development studies where it has resulted in changes to this threshold. Due to the complexity of the concepts involved, the preliminary characterisation of what constitutes a ‘good’ proxy consent decision, and the novelty of developing and evaluating interventions to improve proxy consent decisions, we considered that further rounds of discussion to attempt to reduce the number of items included from 28 was not considered to be fruitful at this stage.

Delphi panel composition may influence results [47]; therefore, we sought to ensure a wide range of stakeholder groups were represented in the Delphi study. However, people with experience of living with an impairing condition were not represented, and only small numbers of people with experience of caring for someone with an impairing condition were included. This was largely due to the impact of COVID-19 as these groups may require additional support to participate. For example, we were unable to visit care homes or dementia support groups to share information about the study as we might have done pre-COVID. As participants were recruited through existing research networks, trials methodology networks, and using social media it is not possible to know how many received an invitation. The timing of the study which was conducted during the coronavirus pandemic may have limited the participation of these groups and presented challenges for clinicians and researchers who were prioritising COVID-19-related clinical care and research. This may have led to different ranking and voting outcomes than would arise from a disparate group with a higher proportion of people with lived experience and may have limited the number of additional outcomes suggested during the Delphi survey rounds.

E-Delphi methods, as necessitated by travel restrictions during the time that this study was conducted, can provide a cost-effective and convenient method of reaching consensus on the items to include in a COS and facilitated stakeholder involvement across a wide geographical area. However, conducting a virtual consensus meeting using an online platform may have limited stakeholders’ ability and willingness to participate in the discussions without the rapport that can be established during face-to-face encounters. Although responses were anonymous to other participants, using online polling may also have influenced voting patterns. As reported in the literature on consensus methods, power differentials between participants and the influence of dominant participants may have affected discussions [48]. Voting outcomes in the early polls may also have influenced voting patterns for subsequent items, and there is the possibility that ‘groupthink’ (the desire for harmony and conformity) could have affected the understanding and decision-making by the group [49]. Polls conducted later in the meeting had more comparable voting patterns than the initial polls, suggesting that members of the group may have developed their understanding about the topic and how to judge and score candidate items.

### Implications for future research

As they were under-represented in this study, further work is needed to explore the views of people living with an impairing condition and their families about the COS items identified. However, due to the complexity of understanding what a ‘good’ proxy decision looks like, and the associated uncertainty about how to measure it, an alternative approach to group consensus discussions will be needed. Additionally, the range of trials involving different populations with impaired capacity are highly heterogenous, consequently proxy decision-making on behalf of these groups and between different types of research may vary. Therefore, further work may also be needed to identify the populations and trials that are most likely to benefit from interventions to enhance proxy participation decisions and where the COS will have the greatest relevance.

Once a COS has been agreed, the next stage in COS development is to determine how the outcomes included in the set should be measured [18]. Several measurement instruments may exist to measure a given outcome, usually with varying psychometric properties (e.g. reliability and validity) [18]. Further work will determine which instruments should be used to measure the identified outcomes using guidance from the COnsensus-based Standards for the selection of health Measurement INstruments (COSMIN) initiative [20]. The list of core outcomes established during the COS will be reviewed alongside the potential outcome measures identified during the scoping review. Further work with a range of stakeholders, including people living with an impairing condition and their families, will be conducted to determine ‘how’ and ‘when’ to measure the outcomes contained in the COS. Any outcomes for which no appropriate instruments or proxy versions of the instrument have been identified will be the target for future development of an appropriate measure. Whilst the relatively long list of outcomes included in the COS may appear burdensome for use in the evaluation of interventions, it is expected that a number of outcome measures (or their sub-scales) will cover several outcome domains and so will not unduly increase participant burden. Further one-to-one work is underway with family members to explore and refine these items, including the feasibility of combining them into a single measure.

## Conclusion

Interventions are increasingly being developed to support aspects of trial conduct, such as consent processes, as part of a growing focus on trials methodology research. Improving trial processes for adults lacking capacity to consent is a previously under-developed area of research. Enhancing the quality of proxy trial participation decisions will ensure that they better reflect the preferences of people who are unable to provide their own consent and reduce the emotional and decisional burden experienced by their proxies. It may also help to address the barriers to including these under-served populations in research, which is the focus of initiatives such as NIHR INCLUDE [50].This study established the items to be included in a core outcome set (COS) for evaluating interventions to enhance the quality of proxy decision-making for research. The findings will be useful to those designing, conducting, and funding research in this new and methodologically developing area, and people living with impairing conditions and their families. Further work is required to identify appropriate measurement instruments, and the relevant timing of outcome measurement, whilst minimising participant burden. Involving a wide range of stakeholders, including people with impairing conditions and their families, in this emerging area of research is essential to ensure that the outcomes are relevant to those populations.

## Supplementary Information

**Additional file 1:** **Appendix 1.** Search strategy.

**Additional file 2:** **Appendix 2.** Candidate outcome items.

**Additional file 3:** **Appendix 3.** Round 2 consensus scores and status.

**Additional file 4:** **Appendix 4.** Consensus scores and status_consensus meeting.

## Data Availability

The anonymised datasets used in the study are available from the corresponding author on reasonable request.

## Abbreviations

COMET: Core Outcome Measures in Effectiveness Trials initiative
COS: Core outcome set
DA: Decision aid
IPDAS: International Patient Decision Aid Standards
PPI: Public and patient involvement
PRISMA: Preferred Reporting Items for Systematic Reviews and Meta-Analyses
RCT: Randomised controlled trial
