# Supplementary material for: Development of a core outcome set for the evaluation of interventions to enhance trial participation decisions on behalf of adults who lack capacity to consent: a mixed methods study (COnSiDER Study)
Source: Trials. 2021 Dec 19;22:935. doi: 10.1186/s13063-021-05883-5 (PMC8684591; doi:10.1186/s13063-021-05883-5)
Supplement: Supplementary file 1 — Additional file 1: Appendix 1. Search strategy. [file 13063_2021_5883_MOESM1_ESM.docx]

**Appendix 1. Search strategy for studies reporting decision aids for proxy decisions**

Ovid MEDLINE(R)

1 Proxy/

2 surrogate.tw.

3 family.tw.

4 ((dementia or alzheimer$ or stroke or critical$) adj7 (family or relative$ or proxy or surrogate$)).tw.

5 1 or 2 or 3 or 4

6 Decision Support Techniques/

7 Decision Support Systems, Clinical/

8 Decision Trees/

9 decision making/

10 Choice Behavior/

11 decision-making computer assisted/

12 ((decision$ or decid$) adj4 (support$ or aid$ or tool$ or instrument$ or techniqu$ or system$ or program$ or algorithm$ or process$ or method$ or intervention$ or material$)).tw.

13 (decision adj (board$ or guide$ or counseling)).tw.

14 (risk communication adj4 tool$).tw.

15 (computer$ adj2 decision making).tw.

16 interactive health communication$.tw.

17 (interacti$ adj4 tool$).tw.

18 6 or 7 or 8 or 9 or 10 or 11 or 12 or 13 or 14 or 15 or 16 or 17

19 randomized controlled trial.pt.

20 controlled clinical trial.pt.

21 randomi?ed.ab.

22 placebo.ab.

23 randomly.ab.

24 trial.ti.

25 19 or 20 or 21 or 22 or 23 or 24

26 5 and 18 and 25
