# Supplementary material for: Development of a core outcome set for the evaluation of interventions to enhance trial participation decisions on behalf of adults who lack capacity to consent: a mixed methods study (COnSiDER Study)
Source: Trials. 2021 Dec 19;22:935. doi: 10.1186/s13063-021-05883-5 (PMC8684591; doi:10.1186/s13063-021-05883-5)
Supplement: Supplementary file 2 — Additional file 2: Appendix 2. Candidate outcome items. [file 13063_2021_5883_MOESM2_ESM.docx]

**Appendix 2. Long list candidate outcome items**

| **How family members/friends make decisions** |
| --- |
| Makes a decision that fits with the person’s own values, wishes, and preferences (values congruence) |
| Is able to determine the person’s own values, wishes and preferences about the choices |
| Is clear about which risks and side-effects would matter most to the person |
| Uses consideration and thought when making the decision or making a choice (deliberation) |
| Is clear about which benefits would matter most to the person |
| Is clear about which would be more important to the person (benefits or risks and side-effects) |
| **Experiences of decision-making in this context** |
| Feels it was the right decision |
| Feels satisfied with the decision |
| Feels that they had enough time to make a decision |
| Feels that the decision made was a good quality decision (i.e how good the decision process was regardless of the outcome) |
| Is comfortable (feels happy and relaxed) with the decision |
| Feels uncertain about the choice made |
| Feels that the decision was a wise one |
| Feels that they have enough support from others to make a decision |
| Feels uncertain about making a decision |
| Has feelings of regret about the way they made a decision |
| Experiences burden of decision-making (e.g time, money, effort) |
| Has feelings of regret about the decision |
| Has feelings of regret about their own role in making the decision |
| Expects that they will stick with the decision |
| Felt it was easy to make |
| **Personal characteristics that influence the decision** |
| Has been informed about the purpose of the research study, procedures, possible risks and benefits |
| Has been informed about their role in making the decision |
| Understands that the person’s own values, wishes, and preferences affect the decision |
| Feels able and has the opportunity to ask questions |
| Feels that they understand the information well enough needed to make the decision (subjective or perceived understanding) |
| Understands the information needed to make the decision (objective understanding) |
| Is ready to make a decision |
| Feels prepared to make the decision |
| Recognises that a decision needs to be made (choice awareness) |
| Feels that they can delay their decision if they feel that they need more time |
| Feels confident in their knowledge to make a decision |
| Feels confident to make a decision |
| Feels able to express their opinion about each choice |
| Feels as involved in the decision as they want to be |
| Recognises the values‐sensitive nature of the decision |
| Recognises they do not have enough information about the views of the individual to represent their views^ |

^ Additional outcome proposed by participants in Round 1

**Additional items proposed by participants in Round 1 but not included in Round 2**

| Makes decision based on the person's wishes and not their own perceptions; beliefs or wishes about research or what it involves |
| --- |
| Attrition rate for the trial - are people changing their mind about the decision they have made on behalf of another |
| Feels confident to acknowledge openly that views of an individual have never been expressed but are acting in Best Interests capacity |
| Whether individuals would want to be consulted at times of stress (i.e. traumatic injury/sudden illness) |
| Whether it is reasonable to expect relatives to read more than 2 A4 sides in emergent situations |
