# Supplementary material for: Development of a core outcome set for the evaluation of interventions to enhance trial participation decisions on behalf of adults who lack capacity to consent: a mixed methods study (COnSiDER Study)
Source: Trials. 2021 Dec 19;22:935. doi: 10.1186/s13063-021-05883-5 (PMC8684591; doi:10.1186/s13063-021-05883-5)
Supplement: Supplementary file 3 — Additional file 3: Appendix 3. Round 2 consensus scores and status. [file 13063_2021_5883_MOESM3_ESM.docx]

**Appendix 3. Round 2 outcome scores and consensus status**

| **Domain - How family members/friends make decisions** | **Participant scores %** | | |  |
| --- | --- | --- | --- | --- |
| **Whether the family members/friend:** | **Not important**  **1-3** | **Important but not critical**  **4-6** | **Critical**  **7-9** | **Consensus status after round 2** |
| Makes a decision that fits with the person’s own values, wishes, and preferences (values congruence) | 5% | 0% | 95% | Include |
| Is able to determine the person’s own values, wishes and preferences about the choices | 0% | 0% | 100% | Include |
| Is clear about which risks and side-effects would matter most to the person | 0% | 15% | 85% | Include |
| Uses consideration and thought when making the decision or making a choice (deliberation) | 0% | 15% | 85% | Include |
| Is clear about which benefits would matter most to the person | 0% | 25% | 75% | Include |
| Is clear about which would be more important to the person (benefits or risks and side-effects) | 0% | 30% | 70% | Include |

| **Domain - Experiences of decision-making in this context** | **Participant scores %** | | |  |
| --- | --- | --- | --- | --- |
| **Whether the family members/friend:** | **Not important**  **1-3** | **Important but not critical**  **4-6** | **Critical**  **7-9** | **Consensus status after round 2** |
| Feels it was the right decision | 5% | 0% | 95% | Include |
| Feels satisfied with the decision | 0% | 20% | 80% | Include |
| Feels that they had enough time to make a decision | 5% | 15% | 80% | Include |
| Feels that the decision made was a good quality decision (i.e how good the decision process was regardless of the outcome) | 10% | 5% | 85% | Include |
| Is comfortable (feels happy and relaxed) with the decision | 5% | 20% | 75% | Include |
| Feels uncertain about the choice made | 0% | 35% | 65% | No consensus |
| Feels that the decision was a wise one | 11% | 20% | 70% | Include |
| Feels that they have enough support from others to make a decision | 0% | 45% | 55% | No consensus |
| Feels uncertain about making a decision | 0% | 60% | 40% | No consensus |
| Has feelings of regret about the way they made a decision | 20% | 5% | 75% | No consensus |
| Experiences burden of decision-making (e.g time, money, effort) | 11% | 65% | 25% | No consensus |
| Has feelings of regret about the decision | 15% | 15% | 70% | No consensus |
| Has feelings of regret about their own role in making the decision | 15% | 30% | 55% | No consensus |
| Expects that they will stick with the decision | 21% | 37% | 42% | No consensus |
| Felt it was easy to make | 20% | 70% | 10% | No consensus |

| **Domain - Personal characteristics that influence the decision** | **Participant scores %** | | |  |
| --- | --- | --- | --- | --- |
| **Whether the family members/friend:** | **Not important**  **1-3** | **Important but not critical**  **4-6** | **Critical**  **7-9** | **Consensus status after round 2** |
| Has been informed about the purpose of the research study, procedures, possible risks and benefits | 0% | 5% | 95% | Include |
| Has been informed about their role in making the decision | 0% | 10% | 90% | Include |
| Understands that the person’s own values, wishes, and preferences affect the decision | 0% | 10% | 90% | Include |
| Feels able and has the opportunity to ask questions | 0% | 5% | 95% | Include |
| Feels that they understand the information well enough needed to make the decision (subjective or perceived understanding) | 0% | 5% | 95% | Include |
| Understands the information needed to make the decision (objective understanding) | 0% | 5% | 95% | Include |
| Is ready to make a decision | 0% | 14% | 86% | Include |
| Feels prepared to make the decision | 0% | 10% | 90% | Include |
| Recognises they do not have enough information about the views of the individual to represent their views^ | 0% | 15% | 85% | Include |
| Recognises that a decision needs to be made (choice awareness) | 0% | 10% | 90% | Include |
| Feels that they can delay their decision if they feel that they need more time | 0% | 19% | 81% | Include |
| Feels confident in their knowledge to make a decision | 0% | 19% | 81% | Include |
| Feels confident to make a decision | 0% | 19% | 81% | Include |
| Feels able to express their opinion about each choice | 0% | 29% | 71% | Include |
| Feels as involved in the decision as they want to be | 0% | 24% | 76% | Include |
| Recognises the values‐sensitive nature of the decision | 0% | 44% | 57% | No consensus |

^ Additional outcome proposed by participant in Round 1
