# Supplementary material for: Development of a core outcome set for the evaluation of interventions to enhance trial participation decisions on behalf of adults who lack capacity to consent: a mixed methods study (COnSiDER Study)
Source: Trials. 2021 Dec 19;22:935. doi: 10.1186/s13063-021-05883-5 (PMC8684591; doi:10.1186/s13063-021-05883-5)
Supplement: Supplementary file 4 — Additional file 4: Appendix 4. Consensus scores and status_consensus meeting. [file 13063_2021_5883_MOESM4_ESM.docx]

**Appendix 4. Consensus scores and status of items considered at the consensus meeting**

| **Item no.** | **Item for discussion** | **Participant scores %*** | | **Consensus status following meeting** |
| --- | --- | --- | --- | --- |
|  |  | **Include** | **Exclude** |  |
| 1 | Experiences burden of decision-making | 33% | 67% | Exclude |
| 2 | Feels that they have enough support from others to make a decision | 92% | 8% | Include |
| 3 | Feels uncertain about making a decision | 8% | 92% | Exclude |
| 4 | Recognises the values‐sensitive nature of the decision | 38% | 62% | Exclude |
| 5 | Feels uncertain about the choice made | 38% | 62% | Exclude |
| 6 | Has feelings of regret about the way they made a decision | 45% | 55% | Exclude |
| 7 | Has feelings of regret about the decision | 64% | 36% | Exclude |
| 8 | Has feelings of regret about their own role in making the decision | 18% | 82% | Exclude |
| 9 | Felt that the decision was easy to make | 55% | 45% | Exclude |
| 10 | Expects that they will stick with the decision | 9% | 91% | Exclude |

* % consensus meeting participants who participated in the poll for that question
